# Biology of Human Cutaneous Melanoma

**DOI:** 10.3390/cancers2010165

**Published:** 2010-03-12

**Authors:** Elias G. Elias, Joanne H. Hasskamp, Bhuvnesh K. Sharma

**Affiliations:** Maryland Melanoma Center, Weinberg Cancer Institute, Franklin Square Hospital Center, Baltimore, MD, USA; E-Mails: joanne.hasskamp@medstar.net (J.H.H.); bhuvnesh.sharma@medstar.net (B.K.S.)

**Keywords:** biology, cutaneous, melanoma, therapeutic approaches

## Abstract

A review of the natural behavior of cutaneous melanoma, clinical and pathological factors, prognostic indicators, some basic research and the present and possible futuristic strategies in the management of this disease are presented. While surgery remains to be the most effective therapeutic approach in the management of early primary lesions, there is no standard adjuvant therapy after surgical resection, or for metastatic disease.

## 1. Introduction

Melanoma consists of a heterogeneous group of tumor cells that vary greatly in their malignant potential. Biologic variability and unpredictable behavior may reflect on its management and end results. We are presenting current clinical and biological facts as well as past and future therapeutic approaches. 

## 2. Clinical-Pathological Facts

### 2.1. Prognostic Factors

Surgery remains to be the only effective therapeutic modality in all types of human melanoma. The wide excision of the primary lesion, elective and therapeutic lymphadenectomy, or sentinel node(s) biopsy to detect early and clinically undetectable lymph node metastases did not change the outcome. Patients’ survival after complete surgical elimination of the detectable disease depends on the stage of the disease and other pathological factors. Some of these factors are related to the host (patient), while others are linked to the disease. 

The host factors that influence patient survival include age, gender, site of primary lesion, and whether the disease developed de novo or in a preexisting nevus [1,2,3]. While there is controversy on each factor, the fact remains that cutaneous melanoma is more common in the third to the sixth decades of life, with predominance at the fifth and sixth decades. Primary lesions of the scalp have a worse prognosis than melanoma arising in the skin of the lower extremity. Women have better survival than men in the first five to seven years. However, most of the primary lesions in women are located on the lower extremities, compared to men whose lesions are more common on the torso. Patients with torso lesions have a poorer outcome. Melanoma of the skin is commonly diagnosed in fair-skin people with light-colored eyes, red to blonde hair and mostly in sun exposed skin. However, it also does occur in dark skin populations, primarily on pale skin areas of the soles of feet, palms of hand, under nails and in mucosa. Although rare, it can originate in the dark skin. The incidence of melanoma in Caucasian to dark skin population is 20:1. A previous personal history of melanoma is considered a risk factor. From a biological point of view, the surgical elimination of the first melanoma lesion does not eliminate the cause. The etiological factors persist and this may result in the development of other primary lesions. For example, a family history of melanoma may indicate that genetic factors could be the culprit. However, even when patients with a family history of melanoma have more than one primary lesion, their overall survival may still be better than a melanoma patient who is the first member in a family with no history of melanoma. Genetic factors may include dysplastic nevi and multiple nevi syndrome with over 50 nevi at one given time. Other nevi that may predispose to skin melanoma include hairy and blue nevi, as well as Hutchinson freckle. DNA mutation may play a major role in the development of melanoma. Xeroderma Pigmentosa is a very rare autosomal recessive skin disorder characterized by deficient DNA repair. These patients have severe solar sensitivity and dry scaly skin with a potential for the development of skin and mucosal cancers including melanoma [4]. 

Several factors at the primary site reflect on the biological behavior of the disease and prognosis. Clark was the first to indicate that the anatomical (histological) level of invasion at the primary site is a prognostic factor [5]. A year later, Breslow reported on the depth of invasion measured in millimeters (mm) at the primary site as a prognostic factor [6]. Breslow’s depth of invasion is the most significant predictor of survival. However, in thin melanoma, i.e., less than 1 mm depth of invasion, Clark level may be a more reliable predictor [5]. In 1986, Clark also was the first to report on vertical growth [7]. It is characterized by the presence of large tumor nests in the papillary dermis. These nests may be rounded or oval and are oriented vertically and perpendicular to the long axis of the epidermis. The cells of the nests have thick chromatin with thick nucleus membranes, which may be notched. These cells may be spindle, small or epithelial and are highly pigmented. Overall the deeper the depth or the level of invasion, the worse is the outcome.

Ulceration of the primary melanoma site carries guarded prognosis. In 1997, we reported on 248 consecutive melanoma patients. We noted that patients with ulcerated primary lesions had poorer outcome, but no statistically significance difference in survival could be reached comparing those with ulcerated lesions to those without ulceration [1]. However, Balch and his colleagues reported on 17,600 melanoma patients and found that patients with ulcerated lesions have statistically significant poorer survival than their counterpart without ulceration [8,9]. Therefore, the American Joint Committee on Cancer (AJCC) staging system for cutaneous melanoma added the ulceration of the primary tumor to the staging system [10]. The biologic reasons behind ulceration are unclear, but it could be that disease progression led to the ulceration. Ulcerated primary lesions seem to have significantly more mitotic cells, another indicator of poor prognosis [11].

An additional biological factor at the primary lesion that may affect the outcome is regression. Regression at the primary melanoma site may signify a role of immune rejection, yet it may give the pathologist false information on the size and depth of the primary lesion. It is characterized by the absence of melanocytic growth in the epidermis and dermis, bordered by melanoma on one or both sides. The epidermis is usually attenuated with loss of reticulum pattern. The dermis is made of non-laminated fibroplasia infiltrated by inflammatory cells and melanophages with telangiectasia, with the vessels arranged perpendicular to the long axis of the epidermis [12]. However, controversy continues on whether regression carries significant risk for metastases [13,14] or not [15,16,17]. The presence of brisk mononuclear infiltrate at the primary site may reflect on better survival [18]. This remains debatable. On very rare occasion since 1960, systemic spontaneous tumor regression has been recognized in patients with wide spread metastases [19]. 

A biological dilemma is metastatic melanoma of an unknown primary. Not all primary melanomas metastasize to regional lymph nodes prior to systemic metastasis. It has been shown that almost 50% of primary lesions do metastasize to lymph nodes, but the other 50% metastasize systemically as the first sign of failure [20]. It is presumed that the primary lesion may have been rejected, but certain tumor cells (clones) survived the rejection and acquired the capability to metastasize. 

Regression of normal skin pigment in the form of vitiligo has also been noted in patients with melanoma regardless of race [21]. Another interesting phenomenon is the presence of acral melanoma in the soles of feet and palms of hands, both in Caucasians and dark skin populations. However, in the dark skin population, such lesions do not cross beyond the black line. Primary melanoma rarely develops in the black skin except very rarely and usually in a pre-existing blue nevus. It is of interest to find mononucleated cells infiltrates at the primary and metastatic lesions, indicating the presence of some immune response [22,23]. However, if such an immune response exists, it seems to be ineffective or insufficient to induce tumor rejection.

When melanoma patients were skin tested with seven bacterial and viral antigens, and their lymphocytes evaluated by in-vitro lymphocyte stimulation assays to three mitogens, their general immunity was not different than normal volunteers. The exception was in patients with end stages of the disease, a time when they become immune suppressed. This could be due to disease load or nutritional reasons. Therefore, it seems that in early stages of the disease, some patients lack recognition of their disease as foreign.

**Figure 1:**
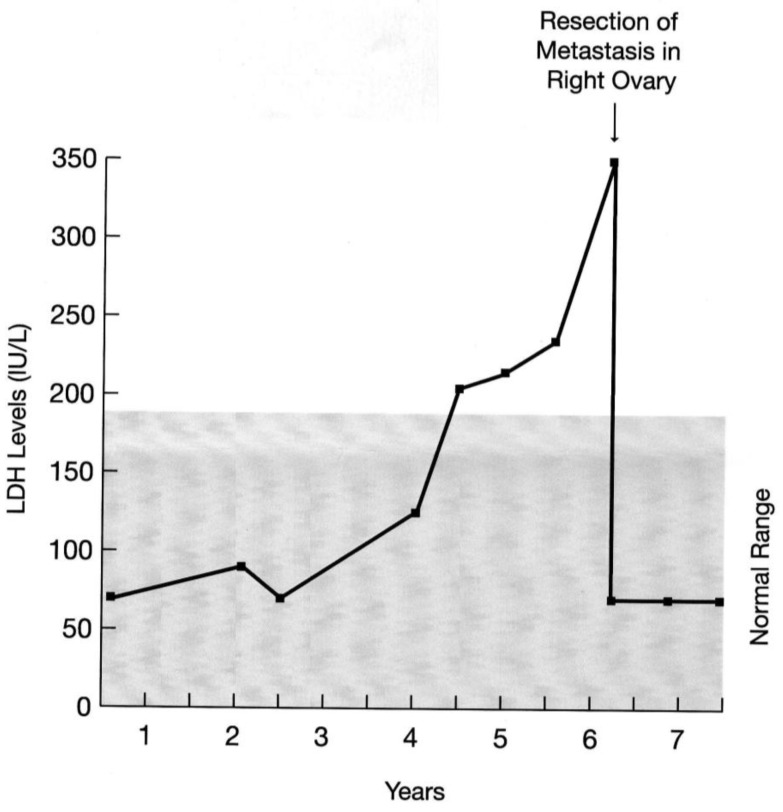
A diagram showing consecutive determinations of serum lactate dehydrogenase (LDH) in a woman with history of resected cutaneous melanoma. Her serial determinations of serum LDH started to rise almost one year before her left ovarian metastasis was diagnosed and resected. Note the precipitous drop in serum LDH level post-operatively. In addition, she received adjuvant autologous vaccine. She is alive and free of disease for over 23 years.

Lactate dehydrogenase (LDH) seems to be secreted by some, but not all, melanoma cells. It has been claimed that elevated levels of serum LDH in melanoma patients carries poor prognosis. However complete resection of metastases can result in the return of LDH levels back to normal and patient’s long survival as seen in Figure 1.

### 2.2. Staging

The American Joint Committee on Cancer (AJCC) classifies melanoma into 4 stages with subclassifications according to number of mitoses per mm^2^, ulceration, nodal status and site of distant metastases (Table 1) [10]. As seen in the AJCC staging system, stages I and II are defined as primary lesions in patients without regional lymph node(s) or distant metastases. The presence of regional lymph node metastases indicates further progression of the disease and is referred to as stage III. To express the extent of metastases in the lymph node, stage III was subclassified to three groups: A, B and C, where A indicates micrometastases in the lymph nodes that are not clinically palpable and are detected only by sentinel lymph node biopsy, B is macrometastases in 1–3 regional lymph nodes and C indicates more tumor burden in the lymph nodes [clinically palpable, enlarged or matted regional lymph nodes].

**Table 1:** AJCC staging system for melanoma (2010).

STAGE	Criteria	Survival Rate	
		5-Year	10-Year
**IA**	**T1a** : No lymph node or distant metastases	97%	93%
	<1mm thickness with no ulceration		
	Number of mitoses <1/mm^2^		
**IB**	**T1b**: No lymph node or distant metastases	94%	87%
	<1mm thickness with ulceration		
	Number of mitoses >1/mm^2^		
	**T2a**: No lymph node or distant metastases	91%	83%
	1–2 mm thickness with no ulceration		
**IIA**	**T2b**: No lymph node or distant metastases	82%	67%
	1–2 mm thickness with ulceration		
	**T3a**: No lymph node or distant metastases	79%	66%
	2–4 mm thickness with no ulceration		
**IIB**	**T3b**: No lymph node or distant metastases	68%	55%
	2–4 mm thickness with ulceration		
	**T4a**: No lymph node or distant metastases	71%	57%
	>4 mm thickness with no ulceration		
**IIC**	**T4b**: No lymph node or distant metastases	55%	39%
	>4 mm thickness with ulceration		
**IIIA**	**N1a**: micro-metastases to one regional lymph node	78%	68%
	without distant metastases		
	**N2a**: micro-metastases to 2–3 regional lymph nodes		
	without distant metastases		
**IIIB**	**N1b**: macro-metastases to one regional lymph node	54%	38%
	without distant metastases		
	**N2b**: macro-metastases to 2–3 regional lymph nodes		
	without distant metastases		
	**N2c**: satellitosis/in transit metastases	59%	43%
	without regional lymph nodal metastases		
**IIIC**	**N3**: 4 or more regional lymph nodal metastases,	40%	24%
	matted regional lymph nodal metastases or		
	satellitosis/in transit metastases with regional nodal metastases		
		**Median Survival**	
**IV**	**M1a**: metastases to skin, subcutaneous tissue or	18 months	
	distant lymph node		
	**M1b**: metastases to lung	12 months	
	**M1c**: metastases to viscera and other sites	6 months	

It is of interest to note that stage IIIA patients had better survival than stage IIC. Stage IIC signifies primary invasive melanoma that is greater than 4mm deep with ulceration but without nodal or distant metastases. Patients with stage IIIA melanoma with micro-metastases in 1 to3 lymph nodes have a 5 and 10 year survival rate ranging from 78% to 68% respectively (Table 1). However, Stage IIC has poorer 5 and 10 year survival rates of 55% and 39% respectively. This could be due to the absence of ulceration at the primary lesion in stage IIIA. While this clearly shows the importance of ulceration of the primary lesion, it may also indicate that early metastases to a functioning regional lymph node could have favorable biological effects. However, the presence of metastases in a lymph node can result in disabling of its immune function.

The worst prognostic feature for melanoma patients is the presence of distant metastases, recognized as stage IV disease. While melanoma can metastasize to any organ, biologically it behaves differently in different metastatic sites. Patients with skin, subcutaneous tissue and distant lymph node metastases had better survival than those with visceral metastases [8]. It has also been shown that several other factors may contribute to the survival of this group of patients. These include the initial stage of the disease, the disease-free interval prior to the development of metastases, the number of metastases and whether these metastases could be resected [24,25,26,27].

At one time, it was thought that melanoma cells were directed to a specific target, i.e., metastatic site. However, it has been shown by Fidler and his colleagues that metastases depend on properties of tumor cells and several host factors [28]. In the early stages of metastases, tumors 2 mm or less in size receive nutrition by diffusion. For such metastases to continue to grow a neo-vascular network has to develop by angiogenesis [29,30,31]. Angiogenesis is mediated by multiple factors which are released by melanoma cells and the host. Among these factors are fibroblast growth factor (FGF), vascular endothelial growth factor (VEGF), epidermal growth factor (EGF), interleukin-8 (IL-8), platelet derived growth factor (PDGF), transforming growth factor-α and-β (TGF-α and-β ), and tumor necrosis factor-α (TNF-α). 

## 3. Clinical Management

### 3.1. Heterogeneity

A major obstacle to an effective treatment is tumor heterogeneity. Melanomas consist of multiple cell populations with various antigens and are capable of secreting a variety of cytokines and growth factors. We have investigated the cytokines and growth factors secreted by human melanoma cell lines and over-expressed in fixed human tissues. Twenty-five cultured melanoma cell lines were studied to measure 14 cytokines and 10 growth factors in the spent medium. Each cell line secreted 5 to 12 cytokines and growth factors simultaneously [32]. In addition, we examined fixed melanoma tissue by immunohistochemistry (IHC) to evaluate the over-expression of cytokines and growth factors at various stages of the disease. The incidence and levels of the cytokines and growth factors varied tremendously within every stage [33]. These findings illustrate the heterogeneity of melanoma. Furthermore, the rate of growth of cutaneous melanoma varies. While some grow fast with a very short latent period from the time of diagnosis to the development of metastases, other melanomas have longer latent periods followed by an accelerated or indolent course. Furthermore, patients with ≥4.0 mm invasive primary, especially with ulceration, are considered at very high-risk of recurrence, metastases and death from the disease, yet some have negative sentinel lymph nodes and have long disease-free and overall survivals.

### 3.2. Surgery and Chemotherapy

Surgery, a local/regional therapeutic modality, remains to be the most effective in the management of this disease. However, the survival results after surgery had reached a plateau. Patients continue to succumb to systemic metastases. Therefore, systemic type of therapy is needed for disease control. Adjuvant therapy is administered to high-risk patients after complete surgical elimination of the disease to prevent recurrence and prolong the overall survival (OS), or at least delay recurrence and prolong disease free survival (DFS). Therapeutic management of patients with unresectable distant metastases is palliative. In general, melanoma failed to show significant response to chemotherapy. Dacarbazine (DTIC) and its precursor temozolomide induced objective tumor response in 5% to 20% of treated patients. Furthermore, extended use of temozolomide failed to improve the outcome [34]. DTIC also failed as adjuvant therapy [35]. 

### 3.3. Cytokine Biotherapy

Spontaneous tumor regression, the presence of immune cells in the form of monocytes in primary as well as in metastatic lesions and the response to bacterial and monoclonal antibodies injected into lesions supported the use of immunotherapy. Biotherapy consisted of three main agents: High-dose interferon alpha-2b (IFN), high-dose interleukin-2 (IL-2) and granulocyte-macrophage colony stimulating factor (GM-CSF). High-dose IFN, an adjuvant therapy approved by US Food & Drug Administration (FDA), had been reported to improve DFS and OS when administered for one-year in high-risk patients post complete surgical elimination of the disease [36]. However, further adjuvant trials with the high dose IFN failed to show these benefits [37]. High-dose IL-2 was also approved by FDA for the treatment of patients with distant metastases based on reported complete responses, some of which were durable [38,39]. Each of these two cytokines has been administered as a single agent, at high doses, over a long period of time. Each resulted in some benefits but also had significant side effects. Each of them has a different mechanism of action with the potential to be synergistic. IL-2 has no direct cytostatic or cytotoxic effect on malignant cells [40]. It modulates the immune reaction in the patients by creating lymphokine-activated killer (LAK) cells and tumor-infiltrating-lymphocytes (TIL) cells. Rosenberg and his associates have shown that lymphocytes from soft tissue metastases are an excellent source for TIL, which are more potent tumor killers than LAK cells when treated with interleukin-2 (IL-2). They also emphasized the significance of autologous TIL with IL-2 therapy [41].

In contrast, IFN has direct and indirect effects. The direct effects include anti-proliferative, pro-differentiating and inhibition of protein synthesis. The indirect effect involves the activation of a variety of host effector cells with secondary production of various cytokines that may result in augmentation of tumor cell surface antigens rendering these cells more susceptible to the host effector cells [42]. Therefore, it was logical to administer low doses of IL-2 and IFN (to minimize toxicity) as adjuvant therapy. In one of our adjuvant trials, we administered low dose IL-2 one week before definitive surgery to activate LAK and TIL cells, and again one week after surgery to magnify such an effect. This was followed one week later by the administration of low dose IFN for one month. The results suggested some survival benefit in the treated patients when compared to historical controls [43]. 

GM-CSF, another immune modulator, was investigated as adjuvant therapy [44,45]. It is less toxic than IFN and the early results were somewhat encouraging. The effect of GM-CSF is mediated through bone marrow stimulation and the production of dendritic cells (DC) and macrophages. Dendritic cells and macrophages are antigen presenting cells (APC) which are rich in the co-stimulatory factors, mainly B7-1 and B7-2. Costimulatory factors are needed for the second signal to complete an immune response. Failure to receive the second signal will lead to clonal anergy. To investigate the effect of the administration of GM-CSF, we studied the levels of mature DC in the peripheral blood immediately after high-dose GM-CSF administration and failed to show any increase in the number of mature DC [46]. Others have reported that patients receiving GM-CSF have shown a transient increase in number of mature DC two weeks after GM-CSF administration [47]. Lineage cocktails and phenotypic markers used to identify DC may differ among various studies resulting in different conclusions. In another trial, we utilized GM-CSF and IL-2 post-operatively as adjuvant therapy and the early results seem to indicate that this combination may be more effective and less toxic than any single agent, especially if combined with autologous melanoma vaccine [48]. However, two reports have cautioned that high-dose GM-CSF can be immunosuppressive [49,50]. This requires further investigation. In the meantime, we have initiated an adjuvant trial utilizing low-dose GM-CSF.

### 3.4. Antibody Biotherapy

When T cells are exposed to an antigen and become activated, two proteins are expressed: CD28 and CD152. CD28 gives a positive stimulatory signal. It is always present on the surface of the T cell and once it receives B7-1 and B7-2 costimulation, it will activate T cell proliferation and IL-2 production. On the other hand, when CD152, also known as cytotoxic T lymphocyte antigen 4 (CTLA-4) is expressed, it gives an inhibitory signal. It is not present on resting T cells, but it becomes upregulated 2–3 days after T cell activation. CD28 and CD152 are closely related proteins with opposing action, theoretically to prevent or minimize over-stimulation and the development of an autoimmune disease. They both compete for the costimulatory factors (B7-1 and B7-2). CTLA-4 binds to B7-1 and B7-2 with much greater affinity than CD28. Since CTLA-4 undermines T cell activation, attempts have been made to block its activity. In a therapeutic approach, anti-CTLA-4 was administered to patients with metastatic melanoma and resulted in tumor regression in 21% of the patients, but 43% developed grade III and IV autoimmune manifestations in the form of dermatitis, enterocolitis, hepatitis, hypophysitis and others [51]. It was interesting to note that higher response rates were detected in patients who developed autoimmune conditions. Combined administration of anti-CTLA-4 and GM-CSF seems to enhance the anti-tumor effect [52]. 

### 3.5. Vaccines

#### 3.5.1. Vaccines of Melanoma Lysates

Immunotherapy with cancer vaccines has explored a variety of approaches. An ideal melanoma vaccine should be constituted of antigens expressed by melanoma cells and not by normal cells. Unfortunately, melanoma antigens are shared by normal cells and are referred to as melanoma associated antigens (MAA). These are peptides, which were identified in the cytosol of melanoma cells and displayed on the cell surface. These peptides bind to class I Major Histocompatibility Complex (MHC) molecules of antigen presenting cells (APC) which process the antigens and transport the information (cross-talk) to T-lymphocytes. Several peptides have been identified and utilized in vaccines. As the peptides originate intracellularly, it was logical to utilize cell lysates to capture these peptides for melanoma specific immunotherapy. Three main types of melanoma cell lysates were evaluated clinically. These were prepared from allogeneic melanoma cells either by viral or mechanical lysing. Viral melanoma oncolysate (VMO) by Wallack was prepared from four melanoma cell lines, potentially containing a variety of MAA, administered as adjuvant therapy to patients with completely resected regional lymph node metastases. It failed to show any benefit in prolonging the disease-free survival (DFS) and overall survival (OS) in a prospective controlled study [53,54]. Vaccinia melanoma cell lysate (VMCL) by Hersey and his colleagues was prepared from viral lysates of a single allogenic cell line and administered as adjuvant therapy but it also failed to show any benefit in a controlled study [55,56]. It was interesting to note that this vaccine contained several MAA including tyrosinase, gp100, MART-1. There is a possibility that the viruses have strong antigenic properties that may have created an immune deviation. The third type of lysate for vaccines was Melacine. Melacine is prepared by mechanical lysing of two allogeneic cell lines and combined with monophosphoryl lipid A and Detox, a purified mycobacterial cell wall (Ribi vaccine). This was administered to patients with invasive melanoma (2–4 mm) with no lymph node metastases. It failed to show any benefit in a prospective controlled study. However, in a retrospective analysis, there was some benefit in a subset of patients with HLA-2A and HLA-C3 with melanoma 3 mm or less depth of invasion [57].

#### 3.5.2. Gangliosides Vaccines

Some melanoma vaccines were designed against expressed surface molecules. Gangliosides are carbohydrate antigens formed of sialic acid containing glycolipid molecules and have increased surface membrane expression on cancer cells of neuroectodermal origin, including melanoma [58]. Melanoma cells are rich in gangliosides; the most prominent are GD3 and GM3 followed by GD2 and GM2. These stimulate a humoral response, producing a short IgM antibody without IgG response [59,60]. To increase the immunogenicity, GM2 was conjugated with keyhole limpet hemocyanin (KLH) and QS-21 to produce higher and consistent IgM response [61,62]. However, a randomized clinical trial comparing IFN for one year to GM2-KLH/QS-21 showed inferiority of the ganglioside vaccine to the IFN [63]. When compared to observation, this vaccine was found to be detrimental [64]. 

#### 3.5.3. Single Peptide Vaccines

The best way to create an immune response is to induce tumor antigens onto APC to process and present to T lymphocytes. Tumor derived proteins, synthetically generated peptides; RNA or DNA are being used as antigen-specific immunizations [65]. Peptide vaccines are usually synthetized rather than genetically engineered, designed to mimic the antigens on melanoma cell membranes that are recognized by cytotoxic T lymphocytes. These MAA were classified into three main categories: 1. Tumor associated testis-specific antigens such as MAGE, BAGE, GAGE; 2. Melanocyte differentiation antigens such as tyrosinase, MART-1 (Melan-1); or 3. Mutated or aberrant expressed molecules including CDK4, MUM-1, β-catinin [66]. These antigens are being evaluated singly or in combinations.

In a clinical adjuvant therapy trial of 25 patients who were immunized with MART 1 with incomplete Freund’s adjuvant (which is wax and oil without BCG), 13 developed positive skin reactions to MART-1 peptide and positive *in vitro* assays for interferon gamma release [67]. However, there was no clinical benefit. Gp100 peptide vaccine was tested as adjuvant therapy in patients with deeply invasive melanoma with or without LN metastases post surgical resection of their tumors [68]. This vaccine induced an increase in the peptide-specific T lymphocytes in the form of a CD8+ cell response. However, as Rosenberg et al pointed out, tumor progression can occur despite the induction of very high levels of tumor specific CD8+ T cells in melanoma patients [69]. MAGE-1 peptide vaccine for immunization of patients with metastatic disease has been reported to induce autologous melanoma-reactive and peptide-specific cytotoxic T cell responses [70]. This was noted at the site of vaccination and at distant tumor sites in patients who were HLA-A1+ and whose tumors expressed MAGE-1 mRNA. MAGE-3 peptide vaccine was administered to 30 patients with metastatic melanoma expressing MAGE-3 gene without the addition of any adjuvant agent. Only one patient expressed an anti-MAGE-3 cytotoxic T-cell response [71].

#### 3.5.4. Multi-Peptides Vaccine

It was thought that the use of multiple peptides could be more effective than the use of a single one. MART-1 + gp100 + tyrosinase were emulsified in incomplete Freund’s adjuvant and administered with progenipoietin, an antagonist to granulocyte colony stimulating factor. It was administered as adjuvant therapy to 15 patients with resected stage III and IV melanoma. Half of the patients developed positive skin response to one or more of the peptides, and 70% demonstrated an immune response to one or more of the peptides by the interferon-gamma release assay. Four of the patients relapsed within a median follow-up of 20 months and one died of the disease [72].

#### 3.5.5. Heat-Shock Peptides Vaccines

Heat-shock peptides are produced by cells under stress, regardless if this stress is physical, chemical or immunological. They are intracellular peptide carriers which are taken up by APC, for presentation to naïve T cells. Autologous tumor derived heat-shock protein gp96 peptide was evaluated in patients with metastatic disease. Two of 28 patients had a complete response for over 3 years [73]. In another report, no survival benefits were noted [74].

#### 3.5.6. Dendritic cell Vaccine

Dendritic cells (DCs) are bone marrow derived cells of the monocyte lineage that can differentiate under the influence of certain cytokines and become mature APC. They ingest tumor antigens, process them, and pass the information to T cells in the context of both MHC class II and I molecules. DCs also express costimulatory factors, B7-1 and B7-2, which are required as the second signal to commit T-lymphocytes to a complete specific immune response. The DCs are very small in number in the peripheral blood. When DC process an antigen, they mature to more effective APC. Patients who were HLA-A2.1+ were vaccinated with mature DCs pulsed with MAA such as gp100 and tyrosinase. They expressed positive delayed hypersensitivity skin reaction to both peptides. This resulted in a complete response (CR) in one out of 26 patients with metastatic disease, but without prolongation of progression-free survival [75]. In another approach, intra-nodal administration of mature DCs did result in T cell stimulation. Twenty seven HLA-A2.1 positive patients with metastatic melanoma were treated with this method, and only one clinical response noted and lasted for over 2½ years [76]. 

#### 3.5.7. DNA Vaccine

Complementary DNA (cDNA) of a specific antigen introduced with a plasmid vehicle and injected in the recipient can be picked-up by APCs such as DCs or other cells such as monocytes and keratinocytes. Clinical trials utilizing DNA-encoding antigens such as tyrosinase, MART-1 and gp100 revealed that repeated administration of this vaccine resulted in the reduction of cytotoxic T lymphocytes responses [77]. 

#### 3.5.8. Viral vaccines

Recombinant poxvirus, adenovirus and other viruses encoding MAA are being investigated [78,79,80]. These approaches attempt to utilize on antiviral response to increase the likelihood of any anti-melanoma associated antigen response.

#### 3.5.9. Allogenic Polyvalent Shed antigens Vaccine:

Bystryn et al introduced a polyvalent vaccine, prepared from three allogenic melanoma cell lines and one xenogenic line that shed antigens in cultured medium [81]. In a prospective controlled study, 38 patients with regional lymph node metastases were randomized after their lymphadenectomy to receive the vaccine versus placebo at 2:1 ratio. The vaccine induced CD8+ T cell responses to gp100, MART-1, MAGE-3 and tyrosinase in 56% of HLA-A01 and HLA-A02 patients. The clinical results revealed a possible increase in disease-free survival of vaccinated patients, but not in overall survival [82,83]. 

### 3.6. Melanoma Cell Vaccines

Most of the tumor antigens recognized by T-lymphocytes are still unknown, and therefore, tumor cells are the best source for total tumor antigens and active specific immunization. Two types of melanoma cell vaccines have been investigated: allogenic melanoma cell vaccine and autologous melanoma cell vaccine. Both were utilized as adjuvant therapy after complete resection of the tumor(s) to reduce the incidence of recurrence.

#### 3.6.1. Allogeneic Cell Vaccines

Allogeneic cell vaccines are constituted of banked tumor cells or cell cultures that are used for active immunotherapy. In 1992, we reported on utilizing allogenic melanoma cells as adjuvant therapy in melanoma [84]. Nine patients with matted metastases to their regional lymph nodes underwent regional lymphadenectomy. Each patient then received a single intradermal (ID) injection of Bacillus Calmette-Guerin (BCG) for sensitization. Three weeks later, vaccination was initiated. Each patient received three vaccinations, each obtained from a different volunteer live donor with wide spread metastases. This was to avoid or minimize the development of a response to HLA antigens while maintaining exposure to melanoma antigens. No cultured cells were used. Each vaccine consisted of mitomycin-c treated melanoma cells mixed with PPD (250TU) given ID once per month for three months. At a median of 5-year follow-up of this small study, 5 patients (56%) were alive and free of disease compared to 40% according to AJCC data for stage III-C (table 1). No autoimmune diseases were encountered in any of the patients.

A polyvalent vaccine, CancerVax, consisted of three cultured cell lines (irradiated and cryopreserved) that were selected for their expression of MAA. It was reported that active specific immunotherapy with CancerVax resulted in delayed type hypersensitivity skin reaction (DHSR) to the MAA and in-vitro cellular immune responses [85]. It also resulted in improved survival in patients with resected lymph node and distant metastases, compared to historical controls [86,87,88]. In addition, most of the benefit with CancerVax was noted in patients who developed DHSR to MAA and had a rise in IgM (TA90). Based on these findings, two randomized adjuvant clinical trials were initiated in late 2001 with CancerVax; one recruited patients with resected regional lymph node metastases and the other in resected distant metastases. However, both studies were terminated after an interim analysis revealed lack of evidence for any benefits [89].

#### 3.6.2. Autologous Whole Cell Melanoma Vaccines

The use of autologous whole tumor cell vaccines has two major advantages over any other types of vaccines. It presents all of the antigens of a specific tumor for a specific patient. And it will not stimulate an immune response against allogeneic major histocompatibility (MHC) antigens. The only disadvantage is the limited accessibility of autologous melanoma cells. In 1997, we reported on our first 22 patients who received adjuvant therapy utilizing five sets of irradiated autologous vaccines. Three patients developed recurrences, which were resected, and each of them was revaccinated by tumor cells recovered from the newly resected metastases. At 4 years of follow-up, 15 patients were alive free of disease including the three who were revaccinated [90]. Recurrences after vaccination should not be considered as absolute failure. Such recurrences could be due to the lack of presentation of all tumor cell clones in the initial vaccination, or due to subsequent mutations. Therefore, revaccination with cells from the newly resected metastases should be highly considered. Only patients with wide spread metastases and unresectable disease are not candidates for such active immunotherapy as these patients are loaded with tumor antigen. In another approach, Berd and his colleagues reported on the use of adjuvant therapy in patients with resected regional lymph node metastases with 8 sets of vaccines that consisted of irradiated autologous cells modified by hapten dinitrophenyl (DNP) [91]. In 2004, they reported 214 patients treated in this fashion with an overall survival of 45% [92]. They have also reported that patients who expressed DHSR to their irradiated melanoma cells had better survival than those who did not. 

## 4. Targeted Therapy

### 4.1. Approaches

Targeted therapy approaches of melanoma treatments include those that utilize receptor tyrosine kinases (RTK), transduction pathways, or cytokines/growth factors and their receptors. Agents of targeted therapy may be active against single or multiple targets.

**Figure 2:**
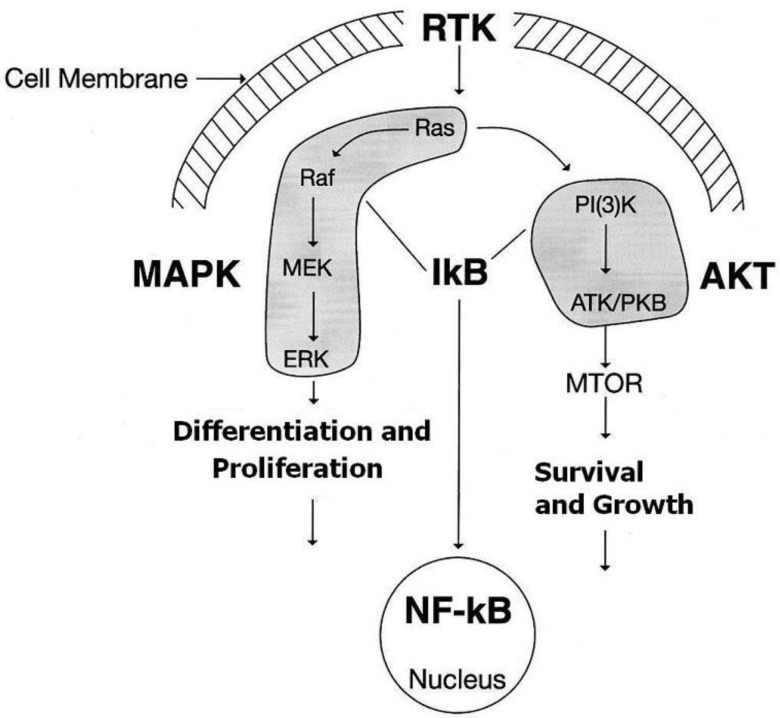
Targeting of the crucial cell signaling pathways of MAPK and AKT has been tested in organotypic skin culture.

Many kinases are the main component of signal transduction pathways that induce cell proliferation and differentiation. Mutation or aberrant expression levels of certain kinases can lead to the development and progression of cancer. Receptor tyrosine kinases (RTK) transmit signals from extracellular to intracellular domains that have been implicated in the development and progression of melanoma [93]. Targeting such kinases was hoped to play a role in inhibiting tumor growth and metastases [94,95]. When transduction pathways become deregulated, it can occur at various stages of cell signaling. This results in the production of excessive factors that act on specific receptors and stimulate tumor cell proliferation. It has been well recognized that melanoma cells express different cytokines and growth factors with their receptors at different stages of the disease. These may act as autocrine and paracrine factors and enable tumor growth and invasion [96]. However, some of these factors expressed by the tumor are also expressed by normal tissue such as the matrix and connective tissue. Crucial cell signaling pathways have been identified in melanoma (Figure 2). These include Ras, Raf, MEK, ERK (MAPK pathway) and PI3k, AKT (AKT pathway) [97]. It has been shown that targeting both MAPK and AKT signaling pathways inhibited melanoma cell growth, survival and invasion in monolayer and organotypic skin culture [98]. However, the clinical applicability of such an approach has not been explored, and the toxicity has not been determined.

### 4.2. Single Targeted Agents

Targeted therapies with a single target have had a variety of approaches. Consider some of the single targeted therapies available today. Vascular endothelial growth factor (VEGF), a potent and specific angiogenic factor, has been identified as a crucial regulator of both normal and pathologic angiogenesis with increased expression in many human tumors [99]. Bevacizumab (Avastin ®) is an anti-VEGF monoclonal antibody. When combined with an effective chemotherapy better response rates occurred in patients with metastatic large bowel cancer. Epidermal growth factor (EGF) and transforming growth factor alpha (TGF-α) stimulate epidermal growth factor receptors, which activate tyrosine kinase and tyrosine phosphorylation with subsequent stimulation of biochemical and physiologic responses involved in mitogenic signal transduction of cells resulting in survival of various cancers [100]. Cetuximab (Erbitux ®) is a humanized monoclonal antibody directed against epidermal growth factor receptor (EGFR). Bcl-2 protein inhibits apoptosis and programmed cell death creating resistance to chemotherapy and radiation therapy. It is expressed in human melanocytes, metastatic melanoma and melanoma cell lines [101]. Oblimersen (Genasense ®) is an anti-sense oligonucleotide that binds to the first 6 codons of human bcl-2 mRNA resulting in degradation of bcl-2 mRNA and decreased protein levels. Histologically, all malignant solid tumors consist of cancer cells in a matrix that contains fibroblasts. Fibroblast activation proteins (FAP) can be targeted for tumor stromal destruction. Talabostat® is a small molecule inhibitor that targets FAP that is present in the stroma of tumors including melanoma. The protein kinase mTOR (mammalian target of rapamycin) is a central component of complex signaling pathways that regulate cell growth and proliferation [102]. Temsirolimus (Torisel®) is a specific inhibitor of mTOR. Cytotoxic T-lymphocyte associated antigen-4 (CTLA-4) is an important regulator of activated T cells. Anti-CTLA-4 monoclonal antibody blocks the interaction of costimulatory factors with CTLA-4 to prolong T cell activation and proliferation [103]. Tremelimumab ® and Ipilimumab® are the two CTLA-4 antagonists that are being evaluated. Except for oblimersen and CTLA-4 antagonists, the single targeted agents described above have been approved for the therapy of cancers other than melanoma. Although melanoma expresses the targets, single agent therapies have been ineffective in significantly improving overall survival. When not effective as single targeted agents, combinations or multi-targeted agents require further study against targets known to be expressed by melanoma. 

### 4.3. Multi-Targeted Agents

Multi-targeted agents used to treat cancer include sunitinib, sorafenib, imatinib, nilotinib, dasatinib and ATN 224. Sunitinib (Sutent®) is a multi-kinase inhibitor targeting several receptor tyrosine kinases, some of them implicated in tumor growth, angiogenesis and metastases. Sunitinib inhibits PDGFα and β, VEGFR, stem cell factor (c-Kit) and others. It is primarily effective in chronic myelogenous leukemia (CML) and gastrointestinal stromal tumors (GIST) [104,105]. Sorafenib (Nexavar®) inhibits Raf, VEGFR-2, 3 and PDGF-β. Combined use of sorafenib and DTIC in patients with metastatic melanoma suggested minimal benefit in progression-free survival that was measured in weeks, but not in overall survival [106]. Imatinib (Gleevec ®) inhibits tyrosine kinases and PDGF. Nilotinib (Tasigna ®) inhibits tyrosine kinases and binds c-Kit and PDGFRA. Both imatinib and nilotinib are effective in the treatment of CML and GIST [107]. Dasatinib (Sprycel®) inhibits tyrosine kinases, PDGFRβ and cKit. It induced some responses in imitanib resistant patients with Philadelphia chromosome-positive acute lymphocytic leukemia (ALL) [108]. All of the above multi-targeted agents have approval for the treatment of either renal cell carcinoma (sunitinib, sorafenib) or leukemia (imatinib, nilotinib, dasatinib) but are also in active clinical trials testing their effect on melanoma. ATN-224 (Attenuon, LLC, SanDiego, CA) binds to copper and has anti-angiogenesis activity. It also down regulates VEGF, FGF2, IL-8 and IL-6 [109]. A trial of ATN-224 and temozolomide in advanced melanoma is ongoing.

## 5. Futuristic Approaches

### 5.1. Adoptive Immunotherapy

Adoptive immunotherapy is the transfer of highly sensitized T cells into patients with metastases after ex-vivo culture of the lymphocytes. Tumor-specific effector cells created by inserting antitumor genes ex-vivo are very efficient in tumor cell kill. Different sources of these cells are being evaluated [110,111].

### 5.2. RNA Interference

Small interfering RNA, siRNA, consists of 20–25 nucleotide long double stranded RNA molecules produced naturally as part of the RNA interference pathway of post-transcriptional gene regulation. It can also be exogenously introduced by various transfection methods to bring about a specific knockdown of a gene of interest. Transfection of exogenous siRNA is transient while transfection with short hairpin RNA, shRNA, produces stable transfectants. Essentially any gene of known sequence can be targeted based on an appropriately tailored siRNA, but off target effects remain unknown. RNAi plays a variety of roles in cancer biology. Tao et al reported that when VEGF-siRNA plasmid was transfected into a melanoma cell line, it resulted in downregulation of the expression of VEGF, inhibition of proliferation, induction of apoptosis in-vitro and suppression of the growth of melanoma *in vivo* [112]. The excessive use of siRNA can result in some side effects due to the activation of innate immune responses and the activation of IFN pathway [113]. 

Similar to siRNA, miRNA is a class of gene regulatory small noncoding RNA. It differs from siRNA as it is processed from single strand RNA precursor with a hairpin structure and is complementary to a part of one or more mRNAs. Some cancers are associated with upregulation and downregulation of certain miRNA genes [114].

### 5.3. Melanoma Stem Cells

The role of stem cells in the development of melanoma is a subject of recent investigations. Realizing that most of cutaneous melanomas arise de novo from normal skin, it will be logic to consider that it has originated from transformed melanocytic stem cells or progenitor cells. Several markers for melanocytic stem cells have been identified including ABCB5, CD20, CD133, CD166 and nestin [115]. These were observed in primary as well as metastatic melanoma [116,117]. Stem cells are capable of self-renewal and differentiation. They also play an important role in chemotherapy resistance, recurrences and the speed of tumor growth. Targeting these tumor-initiating cells could be a strategy for future management of melanoma.

### 5.4. Curcumin

Curcumin is the pigment of the spice turmeric that has shown significant activity *in vitro* by inhibiting the proliferation of early passage human melanoma cell lines [118]. The application of curcumin to melanoma therapy needs further investigation. Obstacles include solubility and bioavailability. There is a need for a solvent other than DMSO or ethanol to dissolve curcumin for safe intravenous use or even intra-arterial use for limb perfusion. Curcumin binds to albumin and becomes less bioactive. Derivatives of curcumin and nanoparticle formulations are being studied to develop more effective doses for systemic treatment.

### 5.5. Radiosurgery

Radiosurgery with steriotactic radiotherapy, also known as “CyberKnife”, has an emerging role in the management of limited metastatic melanoma. It is a radiotherapy machine coupled with a synchrony respiratory tracking system. It can deliver high-dose irradiation over a short period of time with high precision, even to a moving organ. It can be applied to any part of the body. Cyberknife is a type of local therapeutic modality that needs to be supported by an effective systemic therapy. 

## 6. Conclusions

Currently surgery remains the most effective therapeutic modality in the management of melanoma, and the survival of the patients depends on the disease stage at the time of diagnosis. The non-surgical management in the form of adjuvant or therapeutic treatment failed to show any additional impact on survival. The results of chemotherapy and targeted therapy have been disappointing, and none of the lysates, gangliosides, peptides and allogenic whole cell vaccines, singly or in combinations, showed any survival benefits. Allogenic whole cell vaccines probably undergo immune rejection rather than immune sensitization. Autologous whole cell vaccine gave some beneficial results. Recurrences after such vaccination should be resected and the patient revaccinated with the new clones of cells from the newly resected metastases. However, the limited availability of tumor cells and the need for special sterile laboratories for the vaccine preparation are major hurdles.

New therapies under investigation must address the heterogeneity of melanoma. An emerging role of steriotactic radiotherapy as a local therapeutic approach is on the horizon. Targeted therapy utilizing siRNA and melanoma stem cells are futuristic approaches in the management of cutaneous melanoma. Agents such as curcumin that show promise in laboratory testing have the potential to be developed into new therapeutic agents for the treatment of melanoma. 
